# Effect of Boxing Exercises on the Functional Ability and Quality of Life of Individuals with Parkinson’s Disease: A Systematic Review

**DOI:** 10.3390/ejihpe14050085

**Published:** 2024-05-07

**Authors:** Nikolaos Chrysagis, Georgia Trompouki, Dimitris Petropaulis, George A. Koumantakis, Georgios Krekoukias, Georgios Theotokatos, Emmanouil Skordilis, Vasiliki Sakellari

**Affiliations:** 1 Physiotherapy Department, School of Health & Care Sciences, University of West Attica, Agiou Spiridonos 28, 12243 Egaleo, Greece; phys19683163@uniwa.gr (G.T.); phys19683090@uniwa.gr (D.P.); gkoumantakis@uniwa.gr (G.A.K.); gkrekoukias@uniwa.gr (G.K.); 2 Laboratory of Advanced Physiotherapy, Physiotherapy Department, School of Health & Care Sciences, University of West Attica, Agiou Spiridonos 28, 12243 Egaleo, Greece; 3 School of Physical Education and Sport Science, National and Kapodestrian University of Athens, Ethniki Antistaseos 41, 17237 Dapne, Greece; gtheotokatos@phed.uoa.gr (G.T.); eskord@phed.uoa.gr (E.S.)

**Keywords:** Parkinson, boxing exercises, boxing training, functionality, quality of life

## Abstract

Parkinson’s disease (PD) is a neurological disorder caused by the loss of dopamine-producing cells in the substantia nigra and characterized by motor and non-motor symptoms. Boxing is a type of complementary therapy to improve symptoms in PD. The purpose of the present study was to examine the effect of boxing training on the functionality and quality of life of patients with PD. The literature search was performed on PubMed, Scopus, PEDro, Cochrane Library, and Google Scholar search engines. The PEDro scale was used to assess the methodological quality of the studies. This systematic review included three studies that examined disease severity, mobility, physical activity, balance, and quality of life. According to the PEDro scale criteria, the three articles included were of high methodological quality. Statistically significant improvements after the implementation of boxing training was shown for balance and quality of life in contrast to the other variables. Boxing training intervention programs had a positive effect on balance and quality of life in patients with PD; however, the results are conflicting regarding certain functionality variables. Therefore, it is necessary to conduct further research to examine the effectiveness of boxing training on the functionality and quality of life of patients with Parkinson’s disease.

## 1. Introduction

Parkinson’s disease (PD) is a neurodegenerative disorder of the central nervous system (CNS) [1] with an average age of onset of 61 years [2]. It is considered a slowly progressive neurological disorder with motor and non-motor symptoms [3]. Non-motor symptoms can be cognitive, such as impaired time perception, and emotional, such as depression, anxiety, and apathy. In addition, patients may experience disturbances in sleeping, cardiorespiratory, and sensory functions [4,5]. The main motor features of the disease are bradykinesia, rest tremor, muscle rigidity, and postural instability [4]. In addition, postural abnormalities, freezing, speech disorders, and facial expressive mobility may occur [3,6,7,8].

Exercise can improve fitness, gait, posture, and balance [9], and help reduce tremors and bradykinesia in PD patients [10]. Positive effects on the non-motor symptoms of the disease, such as sleep, behavior, and cognitive deficits, have also been reported [10]. According to certain researchers, exercise can increase neuroplasticity and contribute to the regulation of neurotrophic factors that are reduced in PD patients [8,11]. Specifically, exercise may positively impact the brain by improving mitochondrial function, increasing the production of brain-derived neurotrophic factor (BDNF), and cell line-derived neurotrophic factor (GDNF), enhancing synaptic activity and neurogenesis, regenerating angiogenesis, and improving metabolism and glucose utilization [8,10].

Conventional and non-conventional forms of exercise are recommended for the treatment of the symptoms of the disease [12]. Conventional forms of exercise recommended for PD patients may include stretching, treadmill walking, aerobic exercise, and resistance exercises [13]. However, patients may struggle with long-term participation in these exercises due to fatigue and a lack of motivation, especially in a rehabilitation setting [12]. Conversely, non-conventional forms of exercise, such as Tai Chi [14], virtual reality (VR) [15], and dance [16,17], may arouse the interest of patients and maintain their participation in the activities for a long period of time [17]. In particular, Tai Chi is a Chinese martial art that involves slow movements, deep breathing, and relaxation while maintaining various postures and has different styles, such us Chen, Yang, Wu, and Sun [18]. According to a systematic review of systematic reviews and meta-analyses [14], Tai Chi has been found to have a positive impact on the functional mobility and balance of Parkinson’s disease patients. However, the review also notes that, due to the use of various different styles of Tai Chi across the studies, it is difficult to compare and generalize the results. Dance is a creative pursuit that combines aerobic activity and movements that challenge gait and balance with progressively learning motor skills [19]. There is also evidence [16,19] suggesting that dance can be beneficial for PD patients regarding motor function and especially balance. According to the researchers, the limitations of the included studies were that the different styles of dance, such as the tango or Irish dance, and the intensity were not described in detail [16,19]. Furthermore, virtual reality (VR) offers high-intensity, task-oriented, and multi-sensory feedback training [15]. This can include interactive motion-sensing training or exergames games, such as Nintendo Wii Fit and Xbox 360. VR is considered a useful non-conventional therapy that has positive effects on the balance gait and quality of life of PD patients; it is enjoyable, safe, and can be performed at home [15]. On the other hand, people with Parkinson’s disease may face challenges when using virtual reality technology, such as cyber-sickness, cognitive overload, or exercises that are not suitable for their rehabilitation needs [20].

Boxing is another non-conventional form of exercise for PD [11] that usually consists of 2–3 min bursts of intense activity and rest periods [21]. It offers a comprehensive, integrated approach to exercise that covers balance, strength, flexibility, and aerobic exercise. The actions used in boxing incorporate weight-shifting, changing directions, and alternate arm movements that can challenge balance and coordination [22]. Furthermore, it constitutes an effective way to manage both motor and non-motor symptoms of Parkinson’s disease. Unlike walking or running on a treadmill, boxing involves a holistic approach that requires coordination, agility, memory, and quick decision-making skills. Additionally, incorporating cognitive elements, like counting punches and naming colors or animals during training, can be beneficial [23]. This makes it an excellent choice for those with Parkinson’s disease who want to engage in exercises that stimulate both their physical and cognitive abilities [24].

Engaging in boxing as a community-based activity can promote social interaction and be sustainable for an extended period [25]. Participating in team activities can have a positive impact on self-efficacy, foster a supportive environment among team members, and improve team dynamics [25,26]. Adapting exercises based on participants’ ability levels may promote a sense of competence. Furthermore, the variety of activities included in the programs may enhance participant motivation [27].

Boxing for patients with Parkinson’s disease is a non-contact, safe, and accessible exercise with no serious adverse effects [12,24]. Safety can be improved by fostering a direct collaboration between exercise instructors and specialized physical therapists/clinicians, and providing specialized training to the instructors. Specialized trainers can recognize the various symptoms of PD, adjust activities according to the patients’ abilities, and refer them to a physical therapist or neurologist if necessary [24]. Additionally, developing guidelines for necessary modifications and program intensity can enhance safety and feasibility. The adaptation theory of Hutzler [28] may be used as a general framework to manipulate the training variables leading to the expected outcomes. Finally, a client-centered approach using behavioral change theories, such us the Trans-Theoretical Model of Change (TTM) and Self-Determination Theory (SDT), may contribute to the sustainability of the programs and the change in perceptions for the individuals involved [29].

Preliminary non-randomized controlled studies have highlighted the positive effect of boxing programs on the balance [29,30], mobility [31,32,33], and quality of life [34,35] of people with Parkinson’s disease. PD patients who attended boxing programs during the study period reported an improvement in fatigue levels as they felt more energetic, experienced a reduced fear of falling, anxiety, depression, and improved their social lives [36,37]. In addition, they reported a better quality of life than those who had not participated or had previously participated in these programs [26]. High-intensity aerobic exercises incorporated in boxing programs can increase the neurotrophic factor BDNF, reducing damage to dopaminergic neurons in the basal ganglia and improving dopamine production [12]. The factors above suggest that boxing may have a positive impact on motor symptoms in individuals with Parkinson’s disease. Additionally, aerobic training may have a positive effect on depression and sleep through an increase in the release of endorphins, such as serotonin, dopamine, noradrenaline, and basal ganglia function, improving the quality of life of PD patients [37,38]. However, it is important to measure the intensity of boxing programs using subjective evaluation measures, such as a heart rate monitor, to attribute its effect to specific underlying mechanisms [12].

The effectiveness of boxing training, as an adjunctive treatment for PD, has not been adequately investigated to date. Specifically, a systematic review by Morris et al. [12], including a randomized controlled trial and a case series study, as well as a narrative review by Lowery [22], are available in the literature. As Morris et al. [12] noted, the findings were limited due to the small number of studies and participants included. Therefore, it is necessary to systematically combine new evidence with previous research to obtain a more comprehensive understanding of the effectiveness of boxing on various parameters of functionality and quality of life for patients with PD. Thus, the aim of the present systematic review was to investigate the impact of boxing training on the functionality and quality of life of patients with PD incorporating relevant randomized controlled studies.

## 2. Materials and Methods

### 2.1. Study Design

The present systematic review was performed according to the PRISMA method (Preferred Reporting Items for Systematic Reviews and Meta-Analyses) in the new updated version of 2020 [39]. This study has been registered in the PROSPERO international database of prospectively registered systematic reviews in health and social care (CRD42023449007).

### 2.2. Inclusion and Exclusion Criteria

The articles to be included in the present review had to meet the following criteria: (a) Articles were in the English language, (b) randomized controlled trials (RCTs), (c) boxing should have been the method of intervention in at least one of the examined groups, and (d) participants should have been diagnosed with PD. Non-randomized controlled trials, abstracts, and studies in which typical subjects participated were excluded.

### 2.3. Search Strategy

The article search was carried out from April 2023 to October 2023 in the following online databases: PubMed, Scopus, PEDro, Cochrane Library, and Google Scholar. The following keywords were used: Parkinson, Parkinson’s disease, parkinsonism, Parkinson’s disorder, boxing, boxing training, boxing exercise, rock steady boxing, quality of life, and functionality, and their combination, which were obtained according to the PICO method. PICO is an abbreviation of the words Population/Problem Intervention, Comparison, and Outcome, which was used to formulate the clinical question in the literature reviews and to specify the keywords. The four words are the basic elements to formulate a research question according to EBP (evidence-based practice) [40]. PICO components and the search keywords are presented in Table 1. The search was performed using the controlled vocabulary of pre-defined terms (medical subject heading (MeSH) terms) where possible, as well as keywords that showed an association with the variables under consideration. There was no time limit on the search and the results obtained from the electronic databases were thoroughly studied and evaluated by the two investigators (TG and PD).

### 2.4. Data Extraction

Duplicate articles were first removed manually and then articles were reviewed according to their title and abstract. Articles in doubt were further examined by reading the entire content for inclusion or exclusion. Data from the included studies were recorded in tables and included the author, publication year, participant’s characteristics, intervention description (type, frequency, and duration), and outcome data related to functionality and quality of life. Any disagreements that arose between the reviewers were resolved through a discussion until a consensus was reached.

### 2.5. Data Synthesis and Analysis

In the present study, a narrative synthesis was used. Specifically, the merging of the quantitative results between studies was conducted by the vote-counting method [41]. The results are presented according to the outcomes of functionality and quality of life. A meta-analysis could not be conducted as the interventions in the comparison groups and the outcomes in the studies included were not sufficiently similar to ensure a clinically meaningful result [42].

### 2.6. Assessment of Methodological Quality

The methodological quality of the selected articles in the present systematic review was assessed with the PEDro scale. The scale was initially used for randomized controlled clinical trials in physical therapy, while today it has been recognized as a valid and reliable tool for assessing the methodological quality of research in the wider health field [43,44]. More specifically, the scale consists of 11 items (criteria), of which the first concerns external validity, the 2nd–9th items concern internal validity, and items 10–11 are concerned with statistical reports. Each item is answered with yes or no and the maximum score is 10 points. The 1st criterion is related to external validity and is not counted in the overall score [44]. When the total score of the scale exceeds 7/10, it is considered of high methodological quality, while scores 5–6 and 0–4 are considered moderate and poor methodological-quality results, respectively [43,45].

## 3. Results

### 3.1. Search Results

**Figure 1:**
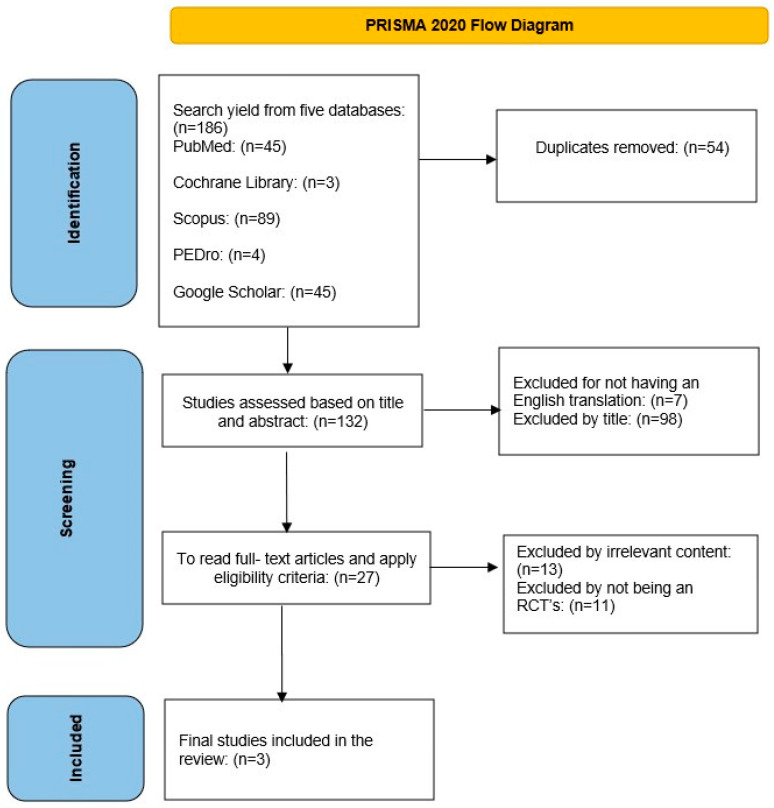
PRISMA 2020 flowchart.

The initial search of the five databases revealed 186 articles according to the defined keywords. Initially, 54 duplicate articles were removed and checked manually. Of the remaining 132 articles assessed, 98 were excluded due to the title or abstract, and 7 more were not translated into English. Of the remaining 27 articles, 13 were not relevant to the content of the systematic review and 11 were not randomized controlled trials. Three articles were finally included in this systematic review. The above is also summarized in the PRISMA 2020 flowchart (Figure 1).

### 3.2. Methodological Quality

The evaluation was performed separately by the two researchers (T.G) and (P.D), and disagreements between the assessors were discussed and resolved through consensus. The three included studies were of a high methodological quality, scoring 7/10 [11,46,47], and the related scores are presented in Table 2.

### 3.3. Characteristics of Included Studies

In the present study, two randomized controlled trials compared the effect of boxing exercises versus other treatments, such as traditional or sensory exercises [11,46]. Another study compared boxing exercises versus boxing with kicking, which in turn constitutes a different form of exercise [47]. The outcome measures in the three randomized trials were mobility, balance, walking ability, and quality of life, with a total of 100 recruited participants with ages ranging from 63.69 to 68 years old. In the study by Combs et al. [46], the patients were at stage 2 [48], with bilateral impairments and without balance decrements, while in the study by Sangarapillai et al. [11], the patients had a score of 2.5 [48] with mild bilateral disease. Domingos et al. [47] did not use a disease severity rating scale and used the ability to walk with assistance as an inclusion criterion. The duration of the interventions was from 10 to 12 weeks, with a frequency ranging from 1 to 3-times per week. Analytical details concerning the intervention programs are presented in Table 3.

### 3.4. Effectiveness of Interventions

#### 3.4.1. Balance

Balance was assessed in two of the three studies [46,47]. Specifically, Domingos et al. [47] assessed balance with the Mini-BESTest after the implementation of the program and found no significant differences between the groups (*p* = 0.53). However, there was an improvement in the boxing group (from 23.09 to 25.80) and in the boxing and kicking group (from 22.60 to 25.33) after the implementation of the programs. Combs et al. [46] who assessed balance with the ABC scale reported significant differences between groups (*p* = 0.015), favoring the traditional exercise group (*p* = 0.022). On the other hand, in the BBS scale, both groups showed a significant improvement before and after the traditional exercise (from 49.0 to 54.0) and boxing (from 49.0 to 53.0) interventions. Proactive balance was assessed in both studies by Combs et al. [46] and Domingos et al. [47], with the TUG (Timed Up and Go) and the dtTUG (Dual-Task Timed Up and Go) tests. There was no significant difference between groups for the above dependent variables. However, Combs et al. [46] reported a significant improvement in TUG (*p* = 0.021) and dTUG (*p* = 0.010) for the traditional exercise program and for the boxing training (TUG (*p* = 0.003) and dTUG (*p* = 0.003)), respectively. On the other hand, a performance reduction was observed for the TUG and dTUG tests for the boxing and kicking and boxing alone groups in the study of Domingos et al. [47]. Table 4 presents the outcome measures and results of the included studies on balance.

#### 3.4.2. Mobility

The effect of boxing training on mobility was assessed, according to the ICF framework [49], with certain walking tests by all three studies [11,46,47]. Combs et al. [46], evaluating mobility through the six-minute test (6 MWT), found a significant improvement (*p* = 0.013) in the distance covered for the boxing group (405.0 to 457.0 m), while no significant improvement was observed (*p* = 0.807) for the traditional exercise group (484.4 to 478.7 m). In the boxing group, there was also a significant difference (*p* = 0.001) and a large effect size (1.46) in walking speed at the end of the intervention (1.06 to 1.10 m/s). Domingos et al. [47] reported no significant differences for each group separately or between groups (*p* = 0.70) at the end of the intervention. Sangarapillai et al. [11] assessed the participants’ stride speed and length in a 10 m walkway (ZenoWalkway-ProtoKinetics). The results showed an improvement mainly for the sensory training group, where the stride length increased from 1.46 to 1.73 m after the end of the program. Sensory training increased stride speed by 0.97 m/s, while the boxing group showed a decrease of 0.08 m/s (*p* < 0.007). The results of the studies on mobility, including the outcome measures, are presented in Table 4.

#### 3.4.3. Quality of Life

Quality of life was assessed with the PDQ-39 questionnaire in two of the three studies [11,47]. In particular, Domingos et al. [47] found no significant difference between the boxing and boxing with kicking interventions employed (*p* = 0.46). However, the researchers reported a statistically significant difference from baseline to the final assessment for the boxing training group (*p* = 0.04) (26.26 to 19.01). Sangarapillai et al. [11] found a significant difference for the main factors of group and time. Specifically, PDQ-39 for the boxing training group improved from 31.4 to 26.20 after 10 weeks of intervention and for the sensory training group from 35.33 to 30.62. However, the rate of improvement recorded was similar for both groups (no significance interaction effect of group × time). In the PDQ-39 questionnaire, the lower the score, the better the quality of life indicating that both interventions improved their overall scores at the end of the program. Finally, Combs et al. [46], comparing boxing training with a traditional exercise program, used the PDQL-37 questionnaire to assess participants’ quality of life. The researchers found an improvement in the quality of life of the participants in both groups at the end of the intervention. More specifically, the traditional exercise group at the initial assessment had a score of 125.5 and after the end of the intervention a score of 149.5. Similarly, the participants of boxing training scored 128.0 at baseline and 132.0 at the end of the study. In the PDQL questionnaire, the higher the final score, the better the quality of life. Outcome measures and results of the included studies on quality of life are presented in Table 4.

**Table 4:** Outcome measures and results of included studies.

Study	Outcomes	Pre	Post	Results Within Groups	Results Between Groups
Combs et al. [46]	TUG (s) Experimental Control	8.05 (15.12) 0.99(0.56)	7.12 (14.62) 1.02(0.61)	*p* = 0.003 *p* = 0.021	*p* = 0.809
	dTUG (s) Experimental Control	11.32 (26.23) 10.33 (16.09)	8.16 (18.24) 8.89 (7.64)	*p* = 0.003 *p* = 0.010	*p* = 0.841
	BBS (total) Experimental Control	49.0 (49.0) 49.0 (17.0)	53.0 (45.0) 54.0 (12.0)	*p* = 0.005 *p* = 0.005	*p* = 0.439
	ABC (%) Experimental Control	83.1 (60.6) 85.0 (56.9)	85.3 (60.6) 93.3 (33.8)	*p* = 0.624 *p* = 0.022	*p* = 0.015
	6 MWT (m) Experimental Control	405.0 (549.1) 484.4 (301.2)	457.0 (669.7) 478.7 (183.9)	*p* = 0.013 *p* = 0.807	*p* = 0.087
	Gait Vel. (m/s) Experimental Control	1.06 (1.08) 1.15 (0.72)	1.10 (1.10) 1.22 (0.64)	*p* = 0.001 *p* = 0.140	*p* = 0.439
	PDQL (total) Experimental Control	128.0 (61.0) 125.5 (84.0)	132.0 (63.0) 149.5 (79.0)	*p* = 0.012 *p* = 0.022	*p* = 0.670
Domingos et al. [47]	TUG (s) Experimental Control	7.74 (2.21) 8.03 (3.05)	8.86 (2.36) 9.14 (2.28)	*p* = 0.007 *p* = 0.06	*p* = 0.72
	TUG dual task (s) Experimental Control	8.46 (2.65) 8.70 (3.17)	9.33 (2.19) 9.65 (2.79)	*p* = 0.23 *p* = 0.07	*p* = 0.72
	Mini-BESTest (total) Experimental Control	23.09 (3.44) 22.60 (2.70)	25.80 (2.39) 25.33 (2.64)	*p* = 0.01 *p* = 0.02	*p* = 0.53
	6 MWD (m) Experimental Control	461.09 (73.63) 467.91 (76.91)	458.40 (67.87) 464.36 (78.07)	*p* = 0.54 *p* = 0.64	*p* = 0.70
	PDQL-39 (total) Experimental Control	26.26 (18.08) 22.52 (12.75)	19.01 (10.62) 25.93 (21.95)	*p* = 0.04 *p* = 0.67	*p* = 0.46
Sangarapi-llai et al. [11]	Stride length (m) Experimental Control	1.48 (0.24) 1.46 (0.13)	1.47 (0.22) 1.73 (0.52)	Not reported	Interaction effect of group × time: F (2, 39) = 5.307, *p* < 0.007
	Stride velocity (m/s) Experimental Control	1.40 (0.17) 1.433 (0.13)	1.36 (0.18) 1.53 (0.20)	Not reported	Interaction effect of group × time: F (2, 39) = 9.825, *p* < 0.0001
	CHAMPS Experimental Control	3149.82 (2040.11) 3844.71 (2963.80)	3146.17 (2059.17) 3847.99 (2483.90)	Not reported	No significant effects or interactions
	PDQ-39 Experimental Control	31.4 (21.97) 35.33 (23.52)	26.20 (30.62) 30.62 (21.75)	Not reported	Significant effect of time: F(2, 39) = 56.533, *p* < 0.0001

## 4. Discussion

The aim of the present systematic review was to investigate the impact of boxing training on the functionality and quality of life of patients with PD. It is noted that this is the first systematic review that includes only randomized controlled trials to examine the effectiveness of that particular intervention. All three selected studies were of high quality. In general, positive results were reported for balance and quality of life, while conflicting results were reported for mobility with all interventions employed.

The present findings are partially in line with the systematic review of Morris et al. [12] and the review of Lowery et al. [22], who reported limited improvement in the mobility and quality of life of people with PD after the implementation of community boxing exercise programs, pointing out that the efficacy of these programs is limited. The above researchers stated that the limited evidence may be due to the small number of participants and the restricted number of randomized controlled trials up to date.

The present findings are also in line with previous systematic reviews examining the effectiveness of other non-conventional treatments on Parkinson’s disease. Specifically, Tai Chi had positive results for balance and quality of life, while no effect was reported for the walking ability of PD participants [14,50]. Carapellotti et al. [16] found that dance had a positive effect on functional mobility and balance, while quality of life improved in only two of the seven studies examined. Lei et al. [15] found that VR training had a positive effect on the balance, gait, and quality of life of PD patients. According to the above researchers, Tai Chi and dance are community-based interventions with the advantage of socialization and the possibility of maintenance of participation [16,24], compared to the VR programs, which are typically carried out at home or in a rehabilitation center, with limited social interaction [20].

Two studies [46,47] from the present systematic review reported significantly improved balance for the participants with PD in the boxing experimental group. In the study of Combs et al. [46], however, the control group that followed a traditional exercise program had better balance performance. According to the researchers [46,47], the results may be due to the different exercises included in the implemented programs. In particular, the typical exercise program included static and dynamic balance activities that simulated activities of daily life, while the boxing program included activities that were not typically related to balance performance. Combs et al. [46] found no significant difference in proactive balance as assessed by the TUG and dtTUG tests between boxing and the traditional exercise groups. However, a significant improvement was observed after the implementation of the program separately for the two groups. Opposite conclusions were reached by Domingos et al. [47] who did not observe an improvement in the performance of PD patients after their participation in boxing programs with or without kicks. In particular, the performance of the patients in proactive balance as assessed by the TUG and dtTUG tests was lower at the end of the program compared to the initial performance. These differences may be related to the duration, timing of the intervention, and the intensity of the program, as in the study by Combs et al. [46] the participants had to perform a minimum of 24 sessions of 90 minutes’ duration for 12 weeks, while the participants in the study by Domingos et al. [47] performed a total of 10 sessions lasting 60 min for 10 weeks. Specifically, a frequency of one session per week may not be adequate to show improvements in the proactive balance of people with PD [34].

Improvements of balance may be related to the activities incorporated in the training program. Specifically, upper-extremity punching motions combined with trunk rotations, anticipatory postural adjustments, and lower-extremity footwork in multiple directions may challenge the visual, somatosensory, and vestibular systems [32,47]. Through the lens of neuroplasticity, high-intensity aerobic exercises incorporated in boxing programs can increase the neurotrophic factor BDNF, reduce the damage to the dopaminergic neurons in the basal ganglia, and improve dopamine production [12,51]. Further engagement in boxing may enhance the functional connectivity of the putamen with the sensorimotor cortex, increase functional connectivity in the right frontoparietal network, and may reduce brain atrophy [52]. Additionally, aerobic exercise is proved to selectively affect specific brain regions on the superior temporal and parietal prefrontal cortexes and transverse tracts between the frontal and parietal lobes and can improve cognitive function in PD [53]. The above factors suggest that boxing may have a positive impact on motor symptoms in individuals with Parkinson’s disease.

Quality of life is reduced in people with PD and constitutes an important factor that must be taken into account when evaluating the efficacy of exercise programs in this special population [54]. Quality of life was assessed in all studies in the present systematic review. Specifically, Sangarapillai et al. [11] and Domingos et al. [47] assessed the quality of life with the PDQ-39 questionnaire, while Combs et al. [46] used the PDQL questionnaire after the implementation of boxing exercise protocols. The researchers reported an improvement in self-perceived quality of life, regardless of an improvement in disease severity [11]. This may be due to the fact that the participants improved their physical condition by exercising in a pleasant environment and feeling that they were part of a community [27]. This fact is supported by the study of Larson et al. [26] who conducted an online questionnaire survey for PD patients who participated before or during the study in rock steady boxing (RSB) programs or were simply informed about RSB. The results showed that PD patients attending RSB programs during the study period reported improvements in fatigue, fear of falling, anxiety, depression, and social life. Additionally, they reported a better quality of life compared to those who had not participated or had previously participated in RSB [26]. Boxing programs can increase motivation and participation promoting social interaction and sustained engagement in physical activity [27]. In general, the well-being of people with Parkinson’s disease, which is strongly correlated with the perceived quality of life, may be more important than the clinical outcomes of various interventions and may keep them active at work and in social activities [11,55].

Group exercise programs provide a supportive environment that promotes camaraderie and sharing among the individuals involved [30,37,56]. The participants share their concerns about the progression of the disease, while those with a history of an active involvement in sports may be more supportive to other team members participating in high-intensity activities [56]. Physical condition benefits along with a supportive and pleasant environment may increase motivation for participation and a sense of achievement. The senses of relatedness to others, along with a sense of competence and autonomy are consistent with the psychological needs described in the self-determination theory. When these needs are met, the individuals develop a sense of internal motivation, satisfaction, and well-being, leading them to improve their quality of life [27].

Mobility was assessed in all studies in the present systematic review. Specifically, in the study by Domingos et al. [47], no significant improvement in mobility was observed in any of the two groups that participated in the boxing programs with or without kicking. The above finding may be explained by the fact that the frequency (one session per week) may not have been adequate to show changes in people with PD [34]. The results are in agreement with Sangarapillai et al. [11] who found that the gait parameters (stride length and gait speed) did not improve either in the short-term (after the end of the intervention) or in the long-term (after 10 weeks) periods for the boxing intervention group. In contrast, participants in the sensory integration group significantly improved their stride length and walking speed in the short and long terms [11]. It should, however, be noted that, in the study by Domingos et al. [47], mobility was evaluated with the 6 MWT, while in the study by Sangarapillai et al. [11], mobility was evaluated with the stride length and walking speed on an electronic treadmill instead.

On the other hand, in the study by Combs et al. [46], gait assessed by the 6 MWT and walking speed showed a significant improvement in patients who participated in the boxing group compared to those who participated in the standard traditional exercise program. The different findings between Combs et al. [46] and Domingos et al. [47] may be explained by the frequency of the program employed, the duration of each session, and the respective intensity. In the study by Combs et al. [46], the frequency was 2–3 sessions per week with a duration of 90 min, while Domingos et al.’s [47] frequency was once a week with a duration of 60 min per session. This is in line with the exercise prescription guidelines for people with Parkinson’s disease, which suggest a frequency of 3–5-times per week for aerobic training and 2–3-times per week for resistance, flexibility, or balance training [55]. With respect to intensity, only the study of Sangarapillai et al. [11] used a self-perceived exertion scale, while Combs et al. [46] reported the resting period between boxing exercises and Domingos et al. [47] determined intensity according to the patient’s capacity. In future studies, the intensity of boxing exercises should be described in more detail, specifying, for example, the number of continuous punches during sessions [12]. Heart rate monitors may also be used to record exercise intensity and for safety reasons [12].

High adherence (96%) and retention rates (100%) were reported in the study of Sangarapillai et al. [11]. Similarly, adherence was 85% and retention was 86% in the study of Domingos et al. [47], indicating that boxing is a pleasant and challenging activity for PD patients. It is worth noting that, in the study of Domingos et al. [47], dropouts occurred due to reasons not related to the program, such as moving to another city. On the other hand, there was an increased number of dropouts (n = 9), especially for the boxing group (n = 6) in the study by Combs [46]. However, the majority of the participants in the boxing group reported that they enjoyed the program, and many of the participants in both groups continued exercising after the completion of the study. It is worth mentioning that no fall incidents or adverse effects were reported in the three included studies [11,46,47], indicating that boxing programs are feasible and safe for patients with PD, even the programs that include high-intensity activities [11].

The high adherence and retention rates recorded In the present systematic review suggest that the participants were able to overcome the obstacles and managed successfully to participate. Previous researchers have reported that financial factors, as well as transportation difficulties and accessibility barriers, may be taken into consideration when a boxing program is prescribed to PD patients. Another barrier may be the severity of the disease, which may be a major obstacle for participation. According to researchers [22], only patients at H&Y stages 1–3 may successfully participate. On the other hand, facilitators for participation may include the community nature of the activity that promotes the development of relationships between participants. The emerging relationships, in turn, may increase the adherence and the sustainability of the boxing programs for the patients with PD [57]. It, therefore, appears that community-based boxing interventions and the stages of the patients involved were additional factors contributing to the adherence and retention results recorded for the patients in the present systematic review.

The limited number of randomized studies included in the present systematic review as well as the total number of participants in the studies employed constitute an important limitation that did not allow generalization without caution. Additional limitations include the variability of the boxing programs and outcome measures that make a direct comparison difficult, the lack of a long term follow up that could ascertain the sustainability of the programs, the lack of sub-group comparisons (e.g., PD severity and type of boxing), and patient selection biases in the studies. Τhe exclusion of studies that were not reported in English, the non-inclusion of articles that did not provide free access, and gray literature were also limitations of the present systematic review. Finally, examining only the statistical significance may not capture the clinical significance of the results.

In order to ensure the safety and effectiveness of boxing programs, a collaboration between health professionals and exercise specialists is recommended [24]. Additionally, specialized training of the exercise instructors can contribute to the detection and handling of patients with various symptoms of PD to tailor activities according to the patients’ abilities. According to the adaptation theory, motor behavior is the result of a dynamic interaction between the patient’s capabilities and the physical, social environmental along with the respective barriers or facilitators [28]. Thus, adapting the equipment used, the task structure, the rules, and the instructions provide physical assistance and support from the exercise specialists, which may enhance patient participation. Finally, there is a need for pre-exercise assessment screening for health issues and fall risk of the participants to ensure safety participation in the boxing program [24].

Future RCTs should include a larger number of participants with possible sub-grouping, depending on their symptom severity and patient demographic characteristics (age and gender), examine training programs of a longer duration, examine the maintenance of the results after the end of the intervention (longer term follow-up), as well as perform comparisons with others forms of exercise. The inclusion of more studies could permit a meaningful meta-analysis in the future. The determination of optimal intensity, duration, detailed intervention programs according to participants’ capabilities, as well as the effect of the intervention on specific PD symptoms constitute important issues to examine in the future. Medication reporting, on or off states of medication, as well as the inclusion of participants with different levels of disease progression could provide new insights into the effectiveness of boxing. Finally, determining an effective collaboration between health professionals, exercise specialists, and participants for establishing criteria for participation in vigorous exercise programs, such as boxing, is also an area for future research.

## 5. Conclusions

The aim of the present systematic review was to examine the effectiveness of boxing programs on the functionality and quality of life of PD patients. It constitutes a form of exercise that has been proposed to treat the symptoms of the disease as it combines a set of activities related to balance, flexibility, endurance, and muscle strengthening. Boxing fits into community programs, may enhance the motivation and willingness to participate, and promotes social interaction. The results, although conflicting, highlight the positive effect of boxing programs on quality of life and balance, while conflicting findings are evident with respect to mobility.

## Figures and Tables

**Table 1:** PICO components and the search keywords.

**Population**	“Parkinson” OR “parkinson disease” OR “parkinsonism” OR “parkinson disorder”
**Intervention**	“Boxing” OR “boxing training” OR “boxing exercise” OR “rock steady boxing”
**Comparison**	“Control group” OR “usual therapy” OR “conventional therapy” OR “physiotherapy”
**Outcome**	“Functionality” AND/OR “quality of life”
**Study**	Randomized Controlled Trial

**Table 2:** PEDro scores for the studies included (note: item 1 is not scored).

	Methodological Quality											
Study	1	2	3	4	5	6	7	8	9	10	11	Final Score
Combs et al. [46]	-	1	1	1	0	0	1	0	1	1	1	7/10
Domingos et al. [47]	-	1	0	1	0	0	1	1	1	1	1	7/10
Sangarapillai et al. [11]	-	1	0	1	0	0	1	1	1	1	1	7/10

**Table 3:** Characteristics of included studies.

Study	Age (y) M (SD)	Functional Status (1–5) Hoeh and Yahr	Group E/C	Experimental Group Intervention	Control Group Intervention	Duration	Frequency
Combs et al. [46]	E 68.0 (31.0) C 66.5 (28.0)	Median 2.0 (3.0)	17/14	Boxing. A 15 min warm-up period consisting of various seated exercises, such as multi-planar axial and extremity active range of motion and stretching. Boxing (hitting a variety of punching bags) and endurance exercises (walking, cycling, and running) in a circuit training mode divided into 3 min periods and 1 min rest. Upper-extremity punching motions were combined with trunk rotations, anticipatory postural adjustments, lower-extremity footwork in multiple directions, and agility drills, such us jumping rope. The participants were motivated to undergo intensive training as much as they could handle. The program progressively became more intense encouraging individuals to complete more repetitions per period.	Traditional exercise 15 min warm up period consisting of various seated exercises, such as multi-planar axial and extremity active range of motion. Strength training incorporated exercises for large muscle groups at the upper extremities using self-selected weights and lower extremities using body weight for resistance. Endurance training included walking at a self-selected pace and stair climbing. Static and dynamic balance exercises were performed with eyes open and closed on various surfaces. Participants engaged in activities using discs, rocker boards, or navigating obstacles. Recovery 15 min and breathing exercises.	12 weeks	24–36 sessions (90 min per session)
Domingos et al. [47]	E 64.36 (11.14) C 63.69 (6.63)	Not reported	14/15	Boxing. Warm-up included walking at a variety of speeds, alternating stepping with jab punches, rotating trunk with hook punches, and squatting on upper cuts. Progress from slower to faster punches while performing boxing exercises in front of a mirror (jabs, hooks, uppercuts, and crosses). Punching bag exercises (jabs, hook, uppercuts, and cross). Introducing combinations of punches, such as two jabs, one hook, and two uppercuts. Increase speed and vary the location of punches on the punchbag. Activities, like games, to make exercise fun, e.g., one person stands in front of a bag and the other behind it, and the instructor prompts which arm to use by touching the exerciser’s arm. Mild relaxation with walking and arms circles. Recovery: progressive increase in intensity “according to patient capacity”.	Boxing with kicking. Warm-up included walking with a variety of speeds, alternating stepping with jab punches, rotating trunk with hook punches, and squatting on upper cuts. Boxing exercises in front of a mirror (jabs, hook, uppercuts, and cross) progressing to faster changes and the addition of kick techniques. The same intervention as the control group on punching bag exercises with the difference of adding kicking techniques, weight shifting exercises, and multi-step directions. More exercise combinations every week, and gradually increasing the intensity and speed of the exercises. Mild relaxation can be achieved through simple exercises, such as walking, arm circles, sideways movements with a small kick, and opening both arms wide.	10 weeks	10 sessions 1 session per week (60 min per session)
Sangarapillai et al. [11]	E 64.2 (9.8) C 65.1 (9.2)	2.5	20/20	Boxing: Warm-up, special boxing exercises (high-intensity boxing exercises, shadow boxing, jumping jacks, speedbag drills, and speed exercises). Recovery: progressive increase in intensity for 10 weeks; frequency: 3 times a week	Sensory exercise: warm-up, specific sensory exercises (stretching, walking, and chair exercises) where the participants were encouraged to complete the exercises slowly, in a controlled manner, and with their eyes closed). Recovery.	10 weeks	3 sessions per week 30 sessions (60 min per session)

## Data Availability

Not applicable.
